# Using gait videos to automatically assess anxiety

**DOI:** 10.3389/fpubh.2023.1082139

**Published:** 2023-03-17

**Authors:** Yeye Wen, Baobin Li, Xiaoqian Liu, Deyuan Chen, Shaoshuai Gao, Tingshao Zhu

**Affiliations:** ^1^School of Electronic, Electrical and Communication Engineering, University of Chinese Academy of Sciences, Beijing, China; ^2^Institute of Psychology, Chinese Academy of Sciences, Beijing, China; ^3^School of Computer Science and Technology, University of Chinese Academy of Sciences, Beijing, China; ^4^Department of Psychology, University of Chinese Academy of Sciences, Beijing, China

**Keywords:** anxiety assessment, mental health, gait video, machine learning, reliability and validity

## Abstract

**Background:**

In recent years, the number of people with anxiety disorders has increased worldwide. Methods for identifying anxiety through objective clues are not yet mature, and the reliability and validity of existing modeling methods have not been tested. The objective of this paper is to propose an automatic anxiety assessment model with good reliability and validity.

**Methods:**

This study collected 2D gait videos and Generalized Anxiety Disorder (GAD-7) scale data from 150 participants. We extracted static and dynamic time-domain features and frequency-domain features from the gait videos and used various machine learning approaches to build anxiety assessment models. We evaluated the reliability and validity of the models by comparing the influence of factors such as the frequency-domain feature construction method, training data size, time-frequency features, gender, and odd and even frame data on the model.

**Results:**

The results show that the number of wavelet decomposition layers has a significant impact on the frequency-domain feature modeling, while the size of the gait training data has little impact on the modeling effect. In this study, the time-frequency features contributed to the modeling, with the dynamic features contributing more than the static features. Our model predicts anxiety significantly better in women than in men (*r*_*Male*_ = 0.666, *r*_*Female*_ = 0.763, *p* < 0.001). The best correlation coefficient between the model prediction scores and scale scores for all participants is 0.725 (*p* < 0.001). The correlation coefficient between the model prediction scores for odd and even frame data is 0.801~0.883 (*p* < 0.001).

**Conclusion:**

This study shows that anxiety assessment based on 2D gait video modeling is reliable and effective. Moreover, we provide a basis for the development of a real-time, convenient and non-invasive automatic anxiety assessment method.

## 1. Introduction

The increasing pressure of modern life has led to a decline in global mental health and an increase in anxiety and depression (1). Anxiety disorders are the most common mental health problems worldwide and may cause physiological reactions such as irritability, fatigue, and increased heart rate. A long-term intense anxious state not only affects an individual's social, life, and work responsibilities but also has a serious impact on their physical health (2). Therefore, to improve the mental health of different groups, the demand for mental health services has increased worldwide (3, 4). Fortunately, in recent years, researchers have made new progress in the treatment of mental diseases such as anxiety and depression (5, 6). At the same time, we urgently need to develop a convenient and timely method for assessing anxiety states.

In psychology, the anxiety scale has been carefully designed, revised and tested, and various scale-based assessment methods have been developed (7). Self-reports rely on individuals reporting their symptoms, behaviors, and attitudes (8). At present, self-reports remain the most commonly used and most effective anxiety assessment method (9). However, scale-based assessments have some limitations and are not applicable in some scenarios (10). For example, in scenarios that require multiple measurements, participants completing the same questionnaire multiple times can lead to practice effects (11). In scenarios such as job interviews, scale results may be inaccurate due to social desirability (12). In addition, the self-report method is not suitable for certain populations, such as illiterate or dyslexic individuals. Therefore, we hope to develop more objective indicators to assess anxiety.

Anxiety can affect an individual's physiological responses. Anxious individuals may experience shortness of breath and accelerated heartbeat (2). In addition, fear is a typical symptom of anxiety disorders, and patients may experience muscle tension (13), sweating, trembling (14), and skin conductance and heart rate changes (15). Anxiety-induced fear can also be reflected through facial expressions (16). Giannakakis et al. showed that some specific facial cues, such as eye and mouth movements, are suitable as discriminative indicators of anxiety (17). Anxiety may also be reflected in voice changes. In anxious states, individuals tend to speak quickly at a loud volume (18), showing fewer voice changes and more pauses (19). Gait and anxiety are also related. Gait posture and movement characteristics can indicate a variety of emotions (20, 21). For example, individuals with anxiety tend to pace back and forth (22). Feldman et al. found that compared with healthy people, anxious patients have shorter stride distances and take fewer steps per minute, displaying movement disorders to some extent (23). Other researchers have noted similar characteristics, such as slow gait (24, 25) and balance dysfunction (26, 27). In addition, arm swings, vertical head movements, and lateral upper body swings have also been associated with anxiety (28). Among the various physiological and behavioral characteristics related to anxiety, gait has several advantages, including large variations, non-invasiveness and ease of observation. Thus, gait can serve as an objective indicator for assessing anxiety.

To acquire gait data, some researchers have used body-worn sensors (29), human motion capture systems (30, 31), Kinects (Xbox One Kinect Sensor) (32) and other devices. However, these devices are expensive and complex to operate, which is not conducive to improving the applicability of anxiety assessment methods. In this study, we recorded 2D gait videos using a common camera that is simple to operate, increasing the ease of obtaining data.

In recent years, with the development of machine learning technology, various researchers have used gait to assess anxiety. Jing et al. found that a prediction model based on gait features performed better than a prediction model based on speech features (33). Miao et al. and Zhao et al. established anxiety assessment models, and the correlation coefficients between the anxiety prediction score and the scale score reached 0.4 (34) and 0.51 (35), respectively. Both studies considered the basic statistics of the gait time series data and the amplitude in the frequency domain after a Fourier transform as features. These features are relatively simple, which may increase the make it difficult to express the rich movement characteristics of gait. In addition, these features lack biological or kinematic interpretations. Stark et al. considered five main gait parameters to identify anxiety, namely, the turning angle, neck variance, lumbar rotation, lumbar movement in the sagittal plane, and arm movement (36). Although the above studies established different anxiety assessment models, they did not comprehensively evaluate the model reliability and validity, and did not adequately validate the performance of their models.

In this study, we used 2D gait videos to construct static and dynamic time-domain features and frequency-domain features and established anxiety prediction models through machine learning algorithms. To validate the proposed models, we examined the effects of different frequency-domain feature construction methods, training data sizes and gender on model performance and compared the contributions of different time-frequency features to the modeling results. In addition, we tested the odd-even split-half reliability of the proposed anxiety assessment model. The goal of this study is to provide a convenient auxiliary anxiety assessment method.

The contributions of this study are as follows:

- Build anxiety assessment models using easily accessible 2D gait videos, reducing cost and increasing convenience of anxiety assessment. It was verified that a good anxiety assessment model can be built without using longer gait videos.
- We constructed static and dynamic time-domain features and frequency-domain features with biological kinematic significance, and proved the rationality and necessity of constructing features.
- This study carefully evaluated the performance (validity and reliability) of the anxiety assessment model through experiments. We validated differences in anxiety assessment between men and women, and verified the robustness of our model in a video odd-even split-half test.

The rest of this paper is organized as follows. First, we introduce the research methods and experiments in Section Methods, including the collection and preprocessing of gait data, feature engineering and modeling, and experimental procedures. Then, the results of several comparative experiments are reported in Section Results. A general discussion of the results is given in Section Discussion, explaining the findings of the study and illustrating further work. Finally, concluding remarks is presented in Section Conclusion.

## 2. Methods

In this study, we used a camera to capture participant gait videos (walking back and forth) indoors. The specific gait video collection method is similar to the method described in Wen et al. (37).

After the gait videos were collected, the participants immediately completed a 7-item Generalized Anxiety Disorder (GAD-7) scale assessment. The GAD-7 assessment is a valid and efficient tool for identifying GAD and assessing its severity in clinical practice and research (9). It evaluates anxiety states in the previous 2 weeks and divides anxiety into four levels according to the scale scores, namely, minimal anxiety (0–4), mild anxiety (5–9), moderate anxiety (10–14), and severe anxiety (15–21). The GAD-7 assessment shows good internal consistency (Cronbach α = 0.92) and test-retest reliability (intraclass correlation = 0.83) (9).

Permission for the above protocol was obtained from the Institutional Review Board of the Institute of Psychology, Chinese Academy of Sciences (Approval number: H15010).

**Figure 1:**
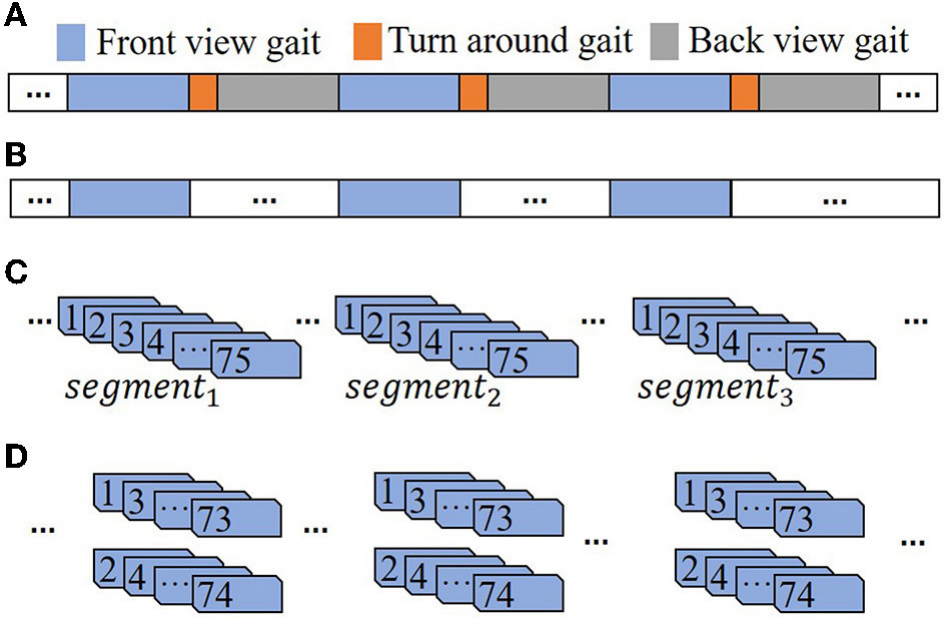
Gait video data segmentation process. **(A)** Full gait video. **(B)** Only the front-view gait segments of the video are kept. **(C)** Keep 75 frames. **(D)** Split odd and even frame segments.

We obtained ~2-min gait videos for each participant, including front and back gaits. Since the front-view gait skeleton evaluation is more accurate than that the back-view evaluation (38), we analyzed skeletons only from the front view to obtain more precise features. Previous studies have shown that good models can be built using a small number of gait frames (35). We kept three consecutive front-view gait segments for each participant, and each segment included 75 frames. To assess the odd-even split-half reliability of the model, we divided the first 74 frames in the gait data into two sets by considering odd and even frames. The gait data segmentation process is shown in Figure 1.

**Figure 2:**
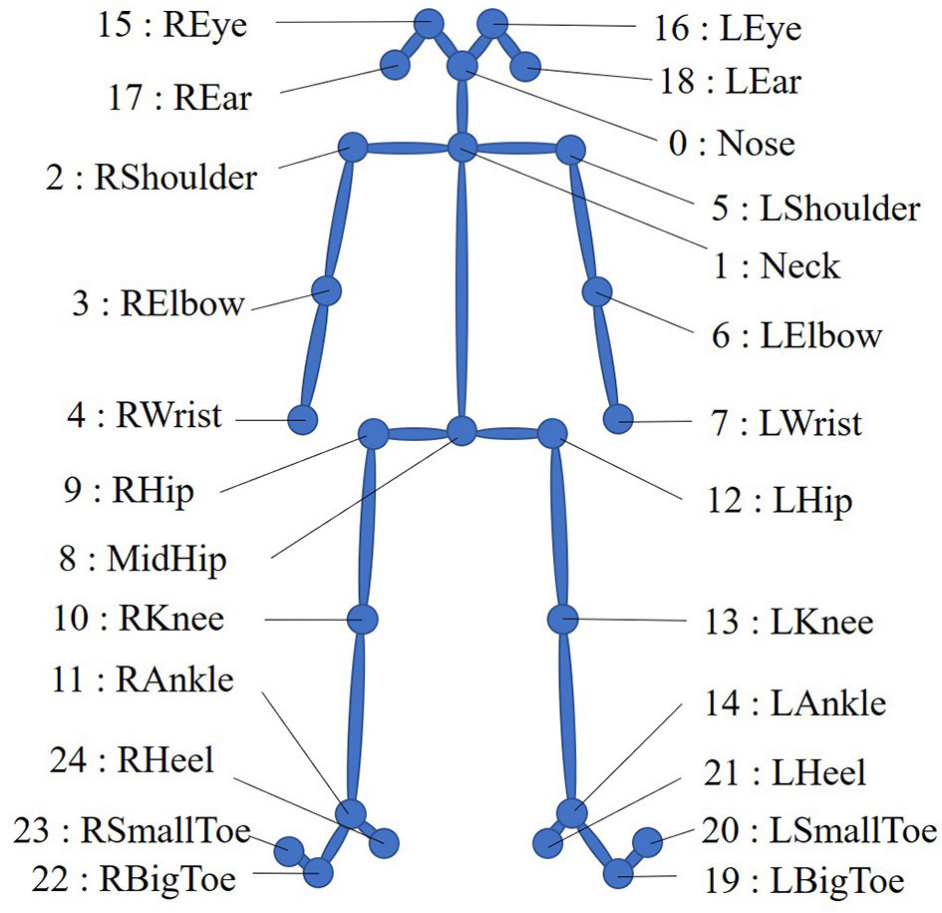
Twenty-five human body key points in OpenPose.

The preprocessing method is similar to the approach proposed in Wen et al. (37). We used OpenPose (39) (a multiperson 2D pose recognition system) to extract the 2D coordinates of 25 body key points from the gait videos and performed coordinate translation (with the MidHip key point as the coordinate origin) and smoothing on the coordinate sequence. Figure 2 shows the 25 human body key points in OpenPose.

**Figure 3:**
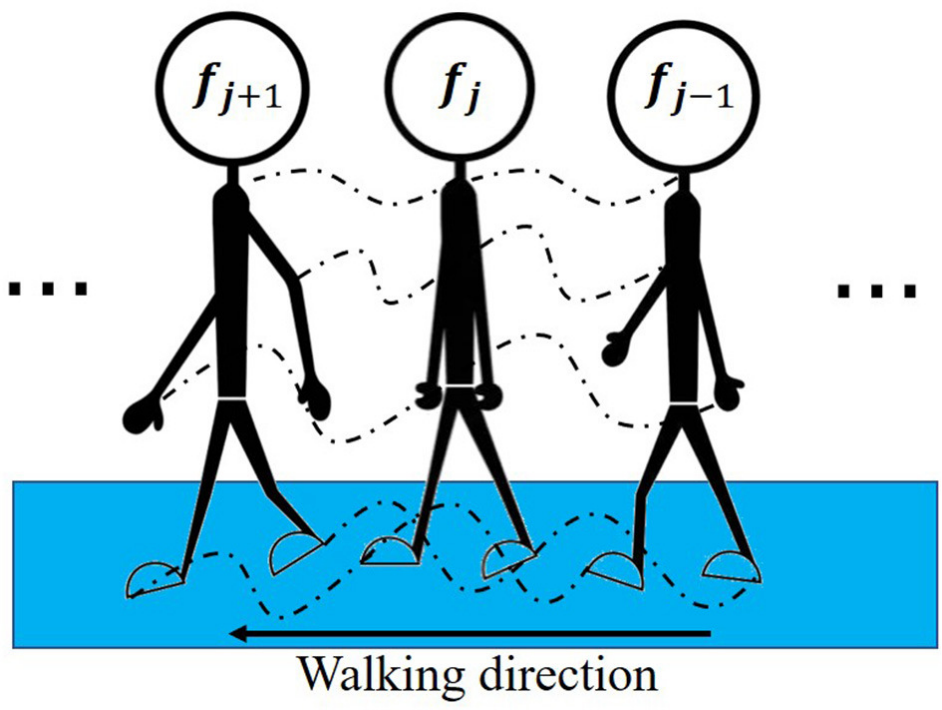
Diagram of the interframe difference. *f*_*j*−1_, *f*_*j*_ and *f*_*j*+1_ represent three adjacent gait images in the gait video. The dotted line represents the movement trajectory of the key point.

The gait coordinate sequence obtained after preprocessing includes only isolated coordinate points and thus does not reflect changes between frames and variations between different key points. We call the features obtained from such data static time-domain features. To reflect the changing gait characteristics (40), we calculate the interframe difference and construct the distances between joints (see Supplementary Table A) and angles between joints (see Supplementary Table B) to express dynamic information. We term these features dynamic time-domain features. The method for obtaining the static and dynamic time-domain features is similar to Wen et al. (37). Figure 3 shows a diagram of the interframe difference between *f*_*j*−1_, *f*_*j*_, and *f*_*j*+1_ in a gait video. The motion track of the key points between each frame contains the interframe difference information.

In gait, some movement patterns are more easily reflected in the frequency domain (41). Relevant studies have extracted frequency-domain gait features through Fourier transforms (34, 35). However, Fourier transforms (42) cannot be applied in multiresolution analyses in the frequency domain. Thus, we use wavelet transforms (43) to analyze the frequency variation characteristics of the joint distances in the frequency domain.

**Figure 4:**
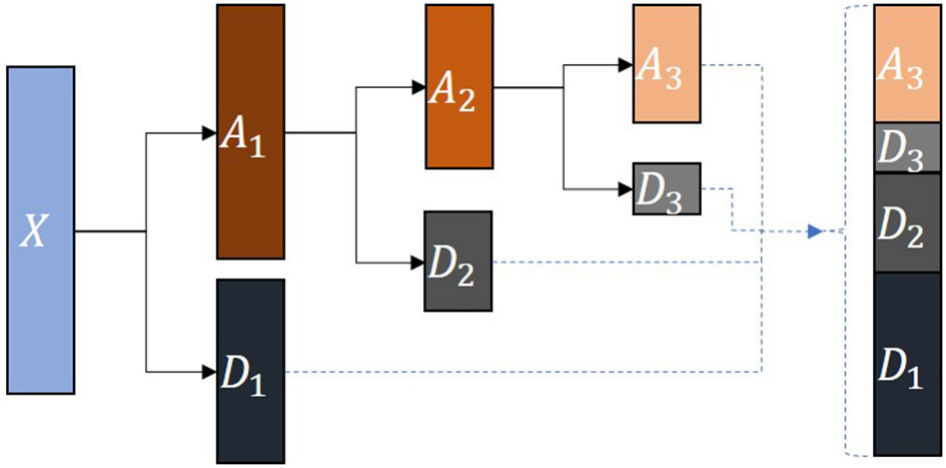
Wavelet decomposition process. *X* represents the source signal. *A*_1_, *A*_2_ and *A*_3_ are the approximation coefficient arrays obtained by decomposing each layer. *D*_1_, *D*_2_ and *D*_3_ are the detail coefficient arrays obtained by decomposing each layer.

We use the *db*1 wavelet base to decompose the distance between joints into an approximation coefficient array *A*_3_ representing low-frequency signals and detail coefficient arrays *D*_1_, *D*_2_, and *D*_3_ representing high-frequency signals. Figure 4 shows the three-layer wavelet decomposition process.

We used 10 feature extraction functions to extract the above time-domain and frequency-domain features. These functions include the maximum, minimum, mean, median, variance, root mean square, skewness, kurtosis, absolute energy, and coefficient of variation in the sequence data. The specific feature extraction functions are shown in Supplementary Table C.

We used z-score standardization (44) to eliminate differences in the values and dimensions of features. The z-score standardization is defined as:


$$\begin{array}{l} x^{{}^{\prime }}=\frac{x-\bar{x}}{\sigma_{x}} \end{array}$$

Where $\overset{\bar{}}{x}$ is the sample mean and σ_*x*_ is the sample standard deviation. Then, we used principal component analysis (PCA) (45) to remove redundant features and sequential forward selection (SFS) (46) to automatically identify feature combinations that resulted in optimal model performance. SFS is a greedy search algorithm. At each stage, according to the evaluation rules, the SFS algorithm continuously selects the optimal feature from the remaining features to determine the optimal feature subset. The SFS pseudocode is shown in Algorithm 1.

**Algorithm 1:** Pseudocode for the Sequential Forward Selection algorithm.

**Algorithm:**Sequential Forward Selection. **Input:** *X*: The whole feature set *J*: The model evaluation rules (Using RMSE) **Output:** S: The best subset of features **Method**: (1) Create an empty subset *Z* = {∅} (2) **repeat** (3) Select best remaining feature: $x=\arg\underset{x^{{}^{\prime }}\epsilon Z_{k}}{\min}\left[J\left(Z\;+x\right)\right]$ (4) Update *Z* = *Z* +*x* (5) *S* = *Z* (6) **until** not decreased in *J* OR *Z* = *X*

**Figure 5:**
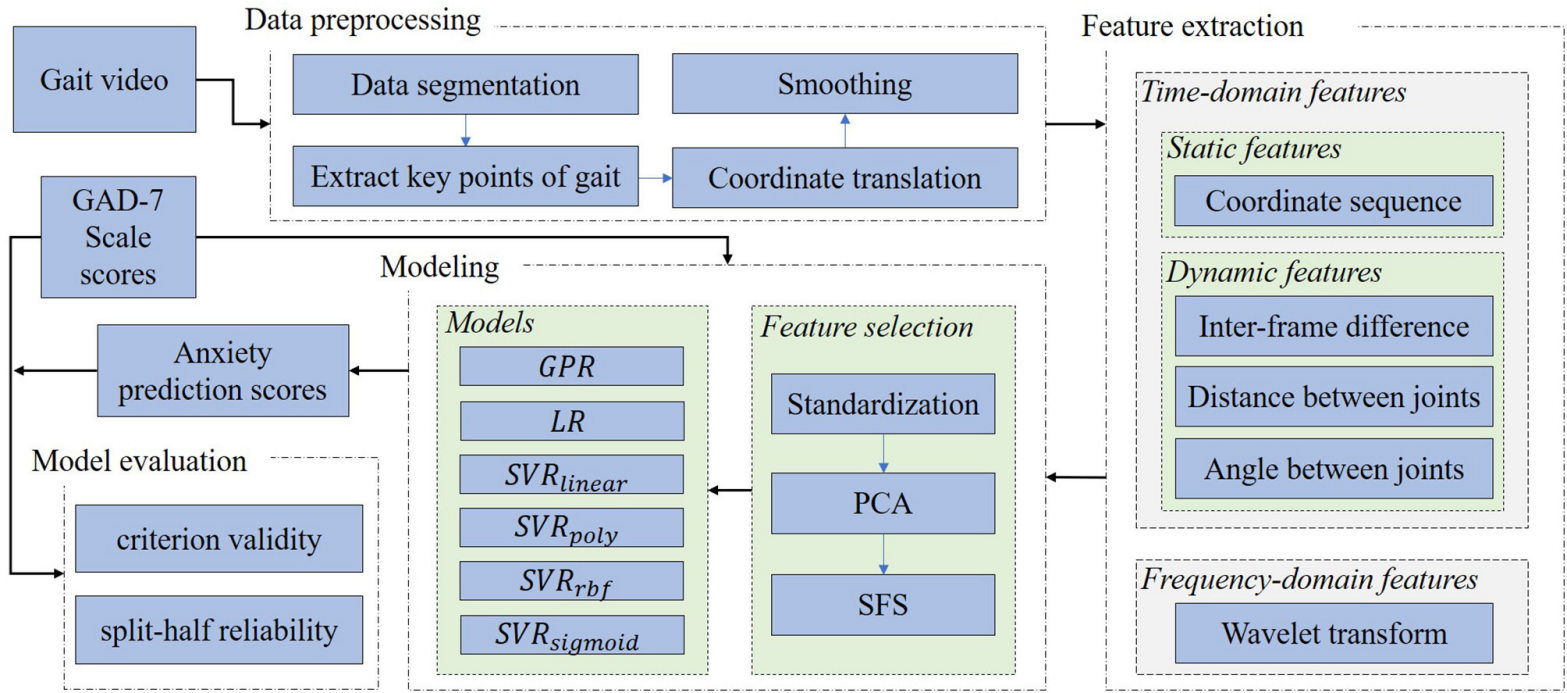
Modeling process. PCA, principal component analysis; SFS, sequential forward selection; GPR, Gaussian process regression; LR, linear regression. *SVR*_*linear*_, *SVR*_*poly*_, *SVR*_*rbf*_, and *SVR*_*sigmoid*_ represent support vector regression using linear, poly, rbf, and sigmoid kernel functions, respectively.

We selected 3 typical machine learning regression algorithms for modeling, namely, Gaussian process regression (GPR), linear regression (LR), and support vector regression (SVR), where the SVR models included the “linear,” “poly,” “rbf,” and “sigmoid” kernel functions. We trained and tested the models with 10 rounds of 10-fold cross validation. The complete modeling process is shown in Figure 5.

In computer science, the root mean square error (RMSE) is often used to evaluate regression model performance (47) and is defined as:


$$\begin{array}{l} RMSE=\sqrt{\frac{1}{N}\overset{N}{\underset{n=1}{\sum }}{\left({Model}_{n}-Scale_{n}\right)}^{2}} \end{array}$$

Where *Model*_*n*_ and *Scale*_*n*_ represent the anxiety model prediction score and anxiety scale score of the nth participant, respectively.

To comprehensively evaluate the performance of the proposed anxiety assessment models, we considered reliability and validity assessment methods used in psychology. We used the Pearson correlation between the anxiety assessment model prediction scores and the anxiety scale scores as the model criterion validity. In addition, we fed different data segments into the model to obtain prediction scores and used the Pearson correlation between these different model prediction scores to evaluate model reliability.

**Figure 6:**
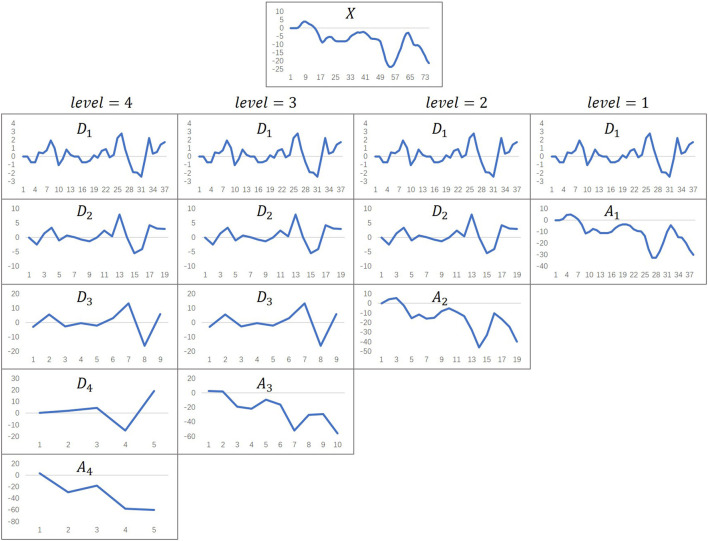
The effect of wavelet decomposition. The level parameter represents the number of wavelet decomposition layers. *X* represents the original time series data. *D*_*i*_ (*i*∈{1, 2, 3, 4}) represent detail coefficient arrays. *A*_*j*_ (*j*∈{1, 2, 3, 4}) represent approximation coefficient arrays.

To explore the influence of the number of wavelet decomposition layers during the construction of the frequency-domain features on the prediction results, we set the wavelet decomposition *level* parameter from 1 to 4 (the *level* parameter controls the number of wavelet decomposition layers). Figure 6 shows the effect of decomposing the original time series signal according to different numbers of wavelet layers. The signals in each column can be restored to the original signal *X* after they are superimposed on each other.

To explore the influence of the gait video training data size on the model, we used gait segments with different numbers of frames to build various models and compared the model performance. In gait data segmentation, each participant has three segments of gait data, as shown in Figure 1. First, we used *segment*_1_, *segment*_2_ and *segment*_3_ to establish three single-segment models. Then, two of the three segments were combined to establish three double-segment fusion models. Finally, the three segments were combined to establish a three-segment fusion model. The gait segments were combined as follows:


$$\begin{array}{l} segment_{12}=segment_{1}+segment_{2}\\ segment_{13}=segment_{1}+segment_{3}\\ segment_{23}=segment_{2}+segment_{3}\\ segment_{123}=segment_{1}+segment_{2}+\;segment_{3} \end{array}$$

The Pearson correlation coefficients between the model prediction scores and the anxiety scale scores were calculated to evaluate the influence of the number of gait segment frames on the performance of the models.

In machine learning, some neural network components can be removed to understand their impact on the network (48). In this study, we explored the impact of different features on model performance through feature ablation studies to determine whether the constructed features are effective. We used the static time-domain features, dynamic time-domain features, all time-domain features (including dynamic and static features), frequency-domain features, and all features (including all time-domain and frequency-domain features) to build 5 anxiety assessment models. The Pearson correlation coefficients between the model prediction scores and the scale scores were used to evaluate the contribution of different features to the model.

We also explored whether gender has an effect on anxiety prediction models. To accomplish this, we input the male and female gait data into the anxiety assessment model. Then, we calculated the Pearson correlation coefficients between the anxiety prediction scores of males and females and the corresponding scale scores to evaluate whether gender impacts the anxiety prediction model.

In psychology, odd-even split-half reliability is often used to characterize the degree of internal consistency of scales (49). We input the odd and even frame gait data into the anxiety assessment model to obtain the corresponding model prediction scores and used the Pearson correlation coefficient between the two prediction scores to evaluate the robustness and reliability of the model.

## 3. Results

We recruited 152 participants. According to the experimental processing requirements, 150 valid data remained after screening, including 79 males (52.67%) and 71 females (47.33%). The proportion of males and females was essentially balanced. The ages of the participants ranged from 21 to 28 years (mean = 22.99, SD = 1.07). The mean and standard deviation of the participant GAD-7 scores were 4.31 and 4.45, respectively. As shown in Table 1, the participants mainly showed minimal and mild anxiety, with 132 participants at this anxiety level (88%). There were 5 participants with severe anxiety, and all were women.

**Table 1:** Population distribution of GAD-7 scale scores.

	**GAD-7 scale score range**				**Total**
	**0**~**4**	**5**~**9**	**10**~**14**	**15**~**21**	
Male	55	18	6	0	79
Female	41	18	7	5	71
Total	96	36	13	5	150

GAD-7, the 7-item Generalized Anxiety Disorder scale; 0~4, minimal anxiety; 5~9, mild anxiety; 10~14, moderate anxiety; 15~21, severe anxiety.

Table 2 show that in terms of the different algorithms, the *GPR* and *LR* models had the best effect, regardless of the number of wavelet decomposition layers. In terms of the number of wavelet decomposition layers, except for the *SVR*_*poly*_ model (the *SVR*_*poly*_ model had the best effect when *level* = 2), the performance of the other models continuously improved as the number of layers increased from *level* = 1 to *level* = 3 (the mean values of *r*_*L*_1__, *r*_*L*_2__ and *r*_*L*_3__ were 0.401, 0.504, and 0.565, respectively). When *level* = 4, the model performance declined (the mean value of *r*_*L*_4__ was 0.464). In summary, the *GPR* and *LR* models showed optimal performance when *level* = 3 (*r*_*L*_3__*GPR*_ = 0.677, *r*_*L*_3__*LR*_ = 0.677, *p* < 0.001, and their *RMSE* values were less than those of the other algorithms). We determined the optimal number of wavelet decomposition layers by iteratively searching parameters.

**Table 2:** Criterion validity of frequency-domain feature modeling using different numbers of wavelet decomposition layers.

	***RMSE*_*L*_1__**	***r*_*L*_1__**	***RMSE*_*L*_2__**	***r*_*L*_2__**	***RMSE*_*L*_3__**	***r*_*L*_3__**	***RMSE*_*L*_4__**	***r*_*L*_4__**
**GPR**	4.027	0.475	3.568	0.594	3.273	0.677	3.830	0.564
**LR**	4.092	0.471	3.593	0.594	3.291	0.677	3.859	0.565
**SVR**_**linear**_	4.024	0.408	3.967	0.430	3.619	0.562	3.946	0.441
**SVR**_**poly**_	4.223	0.269	3.772	0.520	3.915	0.437	4.085	0.409
**SVR**_**rbf**_	4.105	0.405	4.008	0.434	3.967	0.496	4.071	0.390
**SVR**_**sigmoid**_	4.045	0.375	3.952	0.451	3.773	0.542	3.988	0.416

The subscripts *L*_1_, *L*_2_, *L*_3_ *and* *L*_4_ indicate that the numbers of wavelet decomposition layers are 1, 2, 3, and 4 (the *level* parameter ranges from 1 to 4), respectively, when constructing the frequency-domain features. *RMSE* *and* *r* represent the root mean square error and criterion validity of the model established using the frequency-domain features, respectively. All correlation coefficients are highly significant (*p* < 0.001).

As shown in Table 3, among the 7 data combinations, the *GPR* and *LR* models had the best results. In the *GPR* and *LR* models, the modeling effects of the *segment*_1_, *segment*_12_,*segment*_13_ and *segment*_123_ gait segments (which all contained *segment*_1_ and had mean *r*_*s*_1__, *r*_*s*_12__, *r*_*s*_13__ and *r*_*s*_123__ values of 0.559, 0.495, 0.495, and 0.516, respectively) were better than those of the other segments (the mean values of *r*_*s*_2__, *r*_*s*_3__ and *r*_*s*_23__ were 0.425, 0.435, and 0.447, respectively). Similar trends were found for the *SVR*_*rbf*_ and *SVR*_*sigmoid*_ models. In conclusion, the *GPR* and *LR* models had the best performance when modeled on *segment*_1_ (*r*_*s*_1__*GPR*_ = 0.731, *r*_*s*_1__*LR*_ = 0.702, *p* < 0.001). We found that there are some differences in the modeling effect of gait segments in different periods. Moreover, the increase in the number of gait segments did not significantly improve the model effect.

**Table 3:** Criterion validity of modeling with different training data sizes.

	***r*_*s*_1__**	***r*_*s*_2__**	***r*_*s*_3__**	***r*_*s*_12__**	***r*_*s*_13__**	***r*_*s*_23__**	***r*_*s*_123__**
**GPR**	0.731	0.543	0.578	0.633	0.592	0.545	0.634
**LR**	0.702	0.547	0.578	0.630	0.583	0.545	0.637
**SVR**_**linear**_	0.542	0.276	0.320	0.362	0.540	0.426	0.494
**SVR**_**poly**_	0.403	0.386	0.372	0.392	0.314	0.425	0.354
**SVR**_**rbf**_	0.460	0.454	0.403	0.526	0.487	0.425	0.490
**SVR**_**sigmoid**_	0.518	0.346	0.359	0.424	0.454	0.314	0.488

*r*_*s*_1__, *r*_*s*_2__ *and* *r*_*s*_3__represent the criterion validity of the models established using gait segments *segment*_1_, *segment*_2_ and *segment*_3_, respectively. *r*_*s*_12__, *r*_*s*_13__ *and* *r*_*s*_23__ represent the criterion validity of the models established after combining any two of the three gait segments. *r*_*s*_123__ represents the criterion validity of the model established after combining all three gait segments. All correlation coefficients are highly significant (*p* < 0.001).

As shown in Table 4, the modeling effects of the *GPR* and *LR* models on different features were significantly better than those of the other models. The *GPR* model achieved the best modeling effect on all features, including the time-domain and frequency-domain features (*r*_5_*GPR*_ = 0.725, *p* < 0.001). The mean values of *r*_1_, *r*_2_, *r*_3_, *r*_4_, and *r*_5_ were 0.399, 0.446, 0.536, 0.565, and 0.560, respectively, showing a slow increasing trend. These trends were particularly noticeable in the *GPR* and *LR* models, with *r*_5_*GPR*_ > *r*_4_*GPR*_ and *r*_5_*LR*_ > *r*_4_*LR*_ (*p* < 0.001). We found that the anxiety assessment models are sensitive to different gait features. And gait features with kinematic characteristics can significantly improve the performance of the model.

**Table 4:** Ablation studies with different modeling features.

	***r*_1_**	***r*_2_**	***r*_3_**	***r*_4_**	***r*_5_**
**GPR**	0.462	0.602	0.681	0.677	0.725
**LR**	0.461	0.595	0.680	0.677	0.704
**SVR**_**linear**_	0.349	0.274	0.498	0.562	0.540
**SVR**_**poly**_	0.410	0.368	0.467	0.437	0.404
**SVR**_**rbf**_	0.378	0.428	0.459	0.496	0.457
**SVR**_**sigmoid**_	0.336	0.407	0.432	0.542	0.528

*r*_1_, *r*_2_, *r*_3_, *r*_4_ *and* *r*_5_ represent the criterion validity of the models developing using static time-domain features, dynamic time-domain features, all time-domain features (including dynamic and static features), frequency-domain features, and all features (including all time-domain and frequency-domain features), respectively. All correlation coefficients are highly significant (*p* < 0.001).

As shown in Table 5, the *GPR* model performed significantly better than the other models (*r*_*All*_*GPR*_ = 0.725, *r*_*Male*_*GPR*_ = 0.666, *r*_*Female*_*GPR*_ = 0.763, *p* < 0.001, and its *RMSE* value was lower than those of the other algorithms). The anxiety prediction effect was better for women than for men (the mean values of *r*_*Male*_ and *r*_*Female*_ were 0.547 and 0.566, respectively). Except for the *SVR*_*linear*_ and *SVR*_*poly*_ models, all other models reflected this characteristic. We found that the prediction performance of anxiety assessment model for different groups is different.

**Table 5:** Criterion validity of the anxiety assessment model for males and females.

	***RMSE***	***r*_*All*_**	***r*_*Male*_**	***r*_*Female*_**
**GPR**	3.185	0.725	0.666	0.763
**LR**	3.430	0.704	0.639	0.722
**SVR**_**linear**_	3.698	0.540	0.632	0.446
**SVR**_**poly**_	4.018	0.404	0.404	0.361
**SVR**_**rbf**_	3.948	0.457	0.469	0.512
**SVR**_**sigmoid**_	3.823	0.528	0.474	0.590

*RMSE*, root mean square error. *r*_*All*_, *r*_*Male*_ *and* *r*_*Female*_ represent the criterion validity of the model for all participants, male participants, and female participants, respectively. All correlation coefficients are highly significant (*p* < 0.001).

As shown in Table 6, except for *SVR*_*poly*_, all models showed good reliability, and their odd-even split-half reliability was > 0.8. This proved the stability of the model to a certain extent. In conclusion, the *GPR* model obtained the best criterion validity and split-half reliability performance.

**Table 6:** The odd-even split-half reliability of anxiety assessment models.

	***GPR***	***LR***	***SVR*_*linear*_**	***SVR*_*poly*_**	***SVR*_*rbf*_**	***SVR*_*sigmoid*_**
**r**_**split−half**_	0.803	0.801	0.808	−0.696	0.876	0.883

*r*_*split*−*half*_ represents the odd-even split-half reliability. All correlation coefficients are highly significant (*p* < 0.001).

Gait-based anxiety assessment methods have not been fully established. Here we migrated our method to a similar dataset (34). The results showed that the GPR model had the best effect. The Pearson correlation coefficient between the predicted scores of the anxiety assessment model and the scale scores reached 0.6, which was higher than the 0.4 reported by Miao et al. (34). In addition, we also tested the odd-even split-half reliability of the model on this dataset to 0.8. This shows that our anxiety assessment model has good robustness.

## 4. Discussion

We demonstrated that automated anxiety assessment using 2D gait videos is feasible. Based on 2D gait videos, we constructed and fused static and dynamic time-domain features and frequency-domain features and used machine learning methods to establish anxiety assessment models. Moreover, we evaluated the criterion validity and split-half reliability of the proposed anxiety prediction models. We also assessed the effects of different frequency-domain feature construction methods, gait training data sizes, and gender differences on the modeling results, verifying the contributions of various time-domain and frequency-domain features. Our results showed that the proposed gait video-based anxiety assessment method had good reliability and validity.

People with anxiety disorders tend to be between 15 and 35 years old (50). Higher education levels appear to have a protective effect on anxiety and depression (51). In our study, the participants ranged from 21 to 28 years old, their educational backgrounds were mainly involved postgraduate education, and their anxiety levels were concentrated between minimal and mild anxiety. This showed that our sample had a certain representativeness in the higher education student groups.

We used the *RMSE* to evaluate the relative performance of different models. Smaller *RMSE* and larger *r* values indicate better model performance. In Tables 2, 4, the *RMSE* and *r* values showed inverse trends. This result showed that it was reasonable to use the criterion validity to evaluate the performance of the models.

As the number of wavelet decomposition layers increases, we can obtain more detail coefficient arrays representing high-frequency information and more approximate coefficient arrays representing low-frequency information. Since our sequence length was 75, the coefficient arrays that cannot be divided into half are filled with zeros in each wavelet decomposition. When the wavelet decomposition level was too high, the length of the coefficient array was too short, and the zero-padding operation introduced more errors, which led to inaccurate frequency-domain features. This was why the mean value of *r*_*L*_4__ was smaller than that of *r*_*L*_3__. Therefore, in wavelet decomposition, as the number of decomposition layers increases, we can more easily distinguish between low-frequency and high-frequency signals. However, the interference errors caused by the continuous subdivision also increase.

In general, in machine learning, more training data leads to better model effects (52). In our experiments, the model performance did not improve and even decreased as the number of gait training segment frames increased. For example, as shown in Table 3, the modeling effect after fusing two or three gait segments was worse than that of single gait segment modeling. On the one hand, gait is a periodic process (53). More gait segments lead to redundant information that does not contribute to modeling. Therefore, it is sufficient to model with fewer gait frames, which is similar to previous research results (34, 35, 37). On the other hand, different gait segments are discontinuous, and directly merging these sequences may cause mutations that reduce model performance to some extent. We also observed that the modeling effect of gait data including *segment*_1_ was better than that of data including other segments, which may be due to the fatigue of participants walking back and forth in a narrow space, which led to inaccuracies in the subsequent gait videos.

Feature ablation studies were performed to examine how different features contribute to modeling. Taking the GPR model with good reliability and validity as an example, *r*_3_*GPR*_>*r*_2_*GPR*_>*r*_1_*GPR*_ verified that gait contains both dynamic and static information and that dynamic information expresses gait characteristics better than static information. Moreover, *r*_5_*GPR*_>*r*_4_*GPR*_ and *r*_5_*GPR*_>*r*_3_*GPR*_ verified that time-domain and frequency-domain information both contribute to modeling. The results of the feature ablation studies showed that the various constructed features were effective and necessary.

Previous studies have shown that the muscular strength of anxious women is significantly lower than that of healthy women and that these two groups show differences in gait, while these differences are not obvious among males (23). In addition, anxiety differs between the genders, and females are more likely to be anxious than males (54). This may be the reason why the anxiety prediction results are better for women than for men. This fact also supports the finding that participants with severe anxiety in Table 1 were all women.

Cronbach's alpha for the GAD-7 scale was 0.92 (9). In general, an alpha value >0.7 is considered to indicate acceptable reliability. In this study, except for the *SVR*_*poly*_ model, the split-half reliability of the models was > 0.8. This result indicates that the odd-even split-half reliability can be applied to evaluate model performance.

This study is a continuation and extension of our previous work (37). We have optimized the methods of data segmentation, frequency-domain feature construction, and feature selection in experiments. Compared with previous studies, we explored in detail the impact of various factors (different features, gait dataset size, gender) on the model through comparative experiments with various parameters. In this study, the modeling method is more objective and reasonable, and the robustness and predictive performance of the anxiety assessment model are improved. Our research has some limitations. During data collection, a single camera was used to capture gait videos of the participants walking back and forth. Thus, the data contained some gait segments (such as turning and back gaits) that were not suitable for modeling. During preprocessing, the segmentation and recombination of different gait segments might introduce data breakpoints that can impact the model effects. In the future, we set the gait data collection scene as participants walking normally on the treadmill, ensuring that only the participants' front-view gait videos are recorded. We will try to avoid damaging the continuity of gait videos in preprocessing. In addition, although we verified the feasibility of assessing anxiety state based on gait videos, the participants were mainly college graduate students. Since this model was trained on only one social group, the generalizability may be insufficient. Thus, we will recruit participants from different groups according to the differences in age, gender, region, culture and economic background to increase the diversity of training data.

Due to the convenience, real-time, and non-invasive properties of our model, our approach can be applied in various scenarios. For example, the model can be applied for personal daily anxiety assessment. Moreover, companies can learn the employee anxiety levels through video data to provide psychological counseling in a timely manner and improve work efficiency. Using this method to assess the anxiety level of social groups in a timely manner can help to improve community mental health and public health. In future work, our proposed method still has some room for improvement. First, our current research uses traditional machine learning models and artificially constructed features. Although we have demonstrated the rationality and effectiveness of the constructed features in experiments, we still rely on a lot of subjective experience in the early stage. In recent years, many studies have made breakthroughs using deep learning (55). So next we will apply deep neural network to automatically extract gait features and train anxiety assessment models with better predictive performance. Second, our current research needs to convert gait video frame by frame into human body key point coordinates, and then calculate and analyze based on these 2D coordinates. In the process of extracting key points, some gait information will be lost, which will affect the model's learning of gait information. In the future work, we will use image streams for modeling directly based on gait video, so that the neural network can capture more detailed information in the gait.

## 5. Conclusion

In this study, we developed a convenient and timely anxiety assessment method that may contribute to improving mental health services. Our experiments show that gait can be used as an objective cue to measure anxiety, the gait video-based anxiety assessment model has good criterion validity and split-half reliability, and the model has a better prediction effect on females than males. In addition, due to the periodicity of gait, increasing the number of gait training segment frames has little effect on the performance of the anxiety assessment model. The results of comparative experiments showed that the static and dynamic time-domain features and frequency-domain features improved model performance. Our preliminary study provides ideas for developing a convenient real-time anxiety assessment method.

## Data availability statement

To protect the privacy of the participants, the original datasets in the article cannot be made public. If necessary, feature datasets of gait are available from the corresponding author on reasonable request. Requests to access the datasets should be directed to TZ, tszhu@psych.ac.cn.

## Ethics statement

The studies involving human participants were reviewed and approved by Institutional Review Board of the Institute of Psychology, Chinese Academy of Sciences. The patients/participants provided their written informed consent to participate in this study.

## Author contributions

YW, BL, XL, and TZ proposed the idea of the research and designed the research method. DC and SG put forward constructive suggestions. TZ and XL provided research data. YW completed the data analysis and modeling and completed the first draft of the manuscript. TZ and SG guided the research process. All authors participated in the editing and reviewing of manuscripts and contributed to the article and approved the submitted version.
