# Supplementary material for: Using gait videos to automatically assess anxiety
Source: Front Public Health. 2023 Mar 17;11:1082139. doi: 10.3389/fpubh.2023.1082139 (PMC10065197; doi:10.3389/fpubh.2023.1082139)
Supplement: Supplementary file 1 [file Data_Sheet_1.PDF]

Supplementary Material

Table A: Distances between joints.

Distance	Meaning
Head swing	The distance between keypoints 1 and 0.
Upper arm swing (right)	The distance between keypoints 3 and 2.
Arm swing (right)	The distance between keypoints 4 and 2.
Upper arm swing (left)	The distance between keypoints 6 and 5.
Arm swing (left)	The distance between keypoints 7 and 5.
Relative swing of both elbows	The distance between keypoints 6 and 3.
Relative swing of both hands	The distance between keypoints 7 and 4.
Thigh swing (right)	The distance between keypoints 10 and 9.
Leg swing (right)	The distance between keypoints 11 and 9.
Thigh swing (left)	The distance between keypoints 13 and 12.
Leg swing (left)	The distance between keypoints 14 and 12.
Relative swing of both knees	The distance between keypoints 13 and 10.
Relative swing of both feet	The distance between keypoints 14 and 11.

$keypoint\ n$ represents the index of the human keypoint in OpenPose.

Table B: Angles between joints.

Angle	Meaning
Neck angle (right)	The angle between keypoints 0, 1 and 2.
Neck angle (left)	The angle between keypoints 0, 1 and 5.
Shoulder angle (right)	The angle between keypoints 1, 2 and 3.
Shoulder angle (left)	The angle between keypoints 1, 5 and 6.
Elbow angle (right)	The angle between keypoints 2, 3 and 4.
Elbow angle (left)	The angle between keypoints 5, 6 and 7.
Hip angle (right)	The angle between keypoints 12, 9 and 10.

Hip angle (left)	The angle between keypoints 9, 12 and 13.
Knee angle (right)	The angle between keypoints 9, 10 and 11.
Knee angle (left)	The angle between keypoints 12, 13 and 14.

$keypoint\ n$ represents the index of the human keypoint in OpenPose.

Table C: Feature extraction functions.

**Feature extraction function**	**Meaning**
maximum (x)	The maximum value of x.
minimum (x)	The minimum value of x.
mean (x)	The mean value of x.
median(x)	The median of x.
variance (x)	The variance of x.
root_mean_square(x)	The root mean square of x.
skewness(x)	The skewness of x.
kurtosis(x)	The kurtosis of x.
abs_energy(x)	The absolute energy of x.
variation_coefficient(x)	The coefficient of variation of x.

x represents sequence data.
